# Physical activity in preclinical Alzheimer disease: current evidence and implications for multimodal longitudinal research

**DOI:** 10.3389/fneur.2026.1978500

**Published:** 2026-10-29

**Authors:** Liang Wang, Wei Lai, Tianqi Wei, Yunbang Zhang, Youliang Wen

**Affiliations:** 1 School of Rehabilitation and Sports Health, Gannan Medical University, Ganzhou, China; 2 Department of Rehabilitation Therapy Teaching and Research, Gannan Health Vocational College, Ganzhou, China; 3 School of Physical Education, Gannan Normal University, Ganzhou, China

**Keywords:** Alzheimer disease, cognitively unimpaired, longitudinal research, multimodal artificial intelligence, physical activity, wearable sensors

## Abstract

Alzheimer disease (AD) pathology may be detectable years before cognitive symptoms, but biomarker testing of cognitively unimpaired people is not recommended for routine clinical use. Objective physical activity (PA), gait, and rest-activity measures are attractive research signals because they are repeatable and relatively inexpensive; however, none are specific to AD. This narrative review examined evidence linking PA, gait, and rest-activity measures to AD-related outcomes and considered their role in multimodal longitudinal research within biomarker-characterized cohorts. Targeted searches of PubMed, Scopus, and IEEE Xplore were conducted from inception through 25 August 2026, and reference lists of pivotal studies and guidance documents were hand-searched. Priority was given to guidelines, longitudinal cohorts, randomized trials, studies using objective behavior measures and AD biomarkers, and externally evaluated prediction models. Observational evidence links higher activity with lower dementia risk, but reverse causation remains plausible. In biomarker-characterized cohorts, greater baseline step count has been associated with slower amyloid-related tau accumulation and slower cognitive and functional decline, not with lower amyloid burden. Rest-activity fragmentation has shown cross-sectional and prospective associations with amyloid-related measures, whereas a 1-year aerobic-exercise trial did not reduce amyloid accumulation. Recent multimodal AI studies demonstrate that non-PET (positron emission tomography) data can estimate amyloid and tau status. Yet, they do not establish the incremental value of free-living PA in cognitively unimpaired community samples. We therefore outline a framework for longitudinal behavioral research in cohorts whose biomarker status has been characterized independently of PA-based selection. Its primary purpose is to examine associations between behavioral measures and subsequent cognitive, functional, and tau trajectories. Whether PA improves individual prediction beyond clinical information and baseline biomarkers remains a separate, unresolved question. PA is considered a contextual behavioral measure, and the framework does not propose using it to determine eligibility for biomarker testing or research recruitment.

## 1 Introduction

Alzheimer disease (AD) is a growing global public-health problem. China is among the countries with the largest absolute number of affected individuals; a national survey estimated that 9.83 million Chinese adults aged 60 years or older were living with AD at the time of assessment (1). Consequently, prevention research has shifted toward the long interval between the emergence of pathology and recognizable cognitive impairment. That interval provides an opportunity for longitudinal investigation before cognitive impairment becomes evident; it does not make every low-cost correlate of dementia a valid test for biological AD.

The distinction matters because amyloid PET, cerebrospinal fluid (CSF), and sufficiently accurate blood-based biomarkers address a biological question, whereas steps, gait, and daily activity rhythms describe behavior and function. The latter may change because of vascular disease, pain, frailty, mood, sleep, medication, social context, or neurodegeneration. Their appeal lies in repeated measurement under everyday conditions, not in pathological specificity.

Multimodal artificial intelligence (AI) can integrate heterogeneous information, but its role depends on the research question. An association between PA and disease progression does not establish that PA improves individual prediction. The latter requires direct comparisons of models with and without PA, evaluated on independent data.

This review has three aims: to summarize human evidence linking PA, gait, and rest-activity measures to dementia risk and AD-specific biomarkers; to distinguish these associations from intervention effects and incremental prediction; and to outline a framework for longitudinal research in biomarker-characterized cognitively unimpaired cohorts.

The central question is whether behavioral variation is associated with subsequent tau, cognitive, or functional change within a defined pathological context. An association between PA and subsequent disease progression does not establish that PA can identify biomarker-positive individuals before testing. Predictive applications therefore require separate evaluation. Multimodal AI is a possible analytical approach, not a required component or an assumed source of benefit.

## 2 Review methods

### 2.1 Search strategy

This narrative review was informed by targeted searches of PubMed, Scopus, and IEEE Xplore from database inception through 25 August 2026. The database queries combined three groups of terms: AD and cognitive status; physical activity (PA), wearable sensing, gait, and rest-activity rhythms; and AI, multimodal analysis, prediction, validation, and cohort enrichment. These queries focused on the intersection of the three topics and were not intended to provide exhaustive coverage of all PA–AD research. Reference lists of pivotal guidance documents and eligible studies were also hand-searched. The database-specific search strings and filters are provided in Supplementary Table S1.

### 2.2 Eligibility criteria

English-language publications, including human studies, relevant reviews, methodological papers, and guidance documents, were considered if they addressed at least one of the following four questions: (i) the biological definition and clinical boundary of preclinical AD; (ii) PA, exercise interventions, gait, or rest-activity measures in relation to dementia, cognition, brain structure, or AD-specific biomarkers, with priority given to objective behavioral measures; (iii) multimodal prediction of amyloid, tau, or clinically relevant progression; or (iv) validation, bias, and implementation of prediction models.

### 2.3 Study selection and data extraction

Titles and abstracts were screened for relevance to the review questions. For studies included in the synthesis, information was extracted on study design, population, exposure measurement, outcome definition, main findings, and interpretive limitations. Study selection was not conducted independently or in duplicate.

### 2.4 Synthesis and appraisal

The evidence was synthesized narratively by intended use and study design. Interpretation considered temporality, measurement specificity, negative findings, transportability, and whether incremental predictive value had been evaluated. Study limitations were discussed qualitatively; no formal study-level risk-of-bias assessment or certainty grading was performed. Independent duplicate screening and meta-analysis were not conducted. The review was prepared with reference to the domains covered by SANRA (2). These methods do not establish exhaustive evidence coverage or the certainty of the overall evidence base. The research proposals are therefore presented as provisional interpretations of the literature.

## 3 Defining preclinical AD and the clinical boundary

The 2024 Alzheimer’s Association criteria define AD biologically and separate the biological stage from the clinical stage (3). Core 1 biomarkers—amyloid PET or sufficiently accurate CSF or plasma measures—can establish the presence of AD neuropathologic change, while Core 2 biomarkers, including tau PET, can add biological staging. Structural magnetic resonance imaging (MRI), gait, retinal measures, inflammatory markers, and wearables may provide useful context, but they are not interchangeable with AD-defining biomarkers.

Terminology is not fully settled. The Alzheimer’s Association framework allows a biological definition for a cognitively unimpaired person but advises against clinical diagnostic testing in that population outside observational or therapeutic research (3). The International Working Group recommends that most biomarker-positive cognitively normal people be described as at risk rather than labeled as having AD, except in more specific presymptomatic circumstances (4). In this review, “preclinical AD” is used only for cognitively unimpaired people with AD-specific biomarker evidence; otherwise, the terms “cognitively unimpaired” or “at risk” are used.

Blood-based biomarkers have improved feasibility but do not eliminate the need for context. In patients with cognitive symptoms undergoing diagnostic evaluation, plasma phosphorylated tau 217 (p-tau217) strategies have shown strong performance (5). The 2025 Alzheimer’s Association guideline conditionally recommends tests with at least 90% sensitivity and 75% specificity for triage and tests with at least 90% sensitivity and 90% specificity as possible substitutes for amyloid PET or CSF within specialized diagnostic care (6). The guideline does not endorse population screening of asymptomatic adults. Assay-specific thresholds, indeterminate zones, comorbidity, pretest probability, and counseling remain material.

Accordingly, this framework is limited to longitudinal research in appropriately consented, biomarker-characterized cohorts. Biomarker acquisition must be justified by the parent protocol independently of any PA-based risk estimate. Behavioral measurements can then be examined in relation to the underlying pathology and subsequent clinical course. The framework does not use PA to determine who should undergo biomarker testing.

## 4 Physical activity and digital motor phenotypes

### 4.1 Dementia risk, temporality, and reverse causation

Physical inactivity is among the potentially modifiable factors identified by the 2024 Lancet Commission on dementia prevention (7). A systematic review and meta-analysis of 58 longitudinal studies reported lower risks of all-cause dementia (pooled relative risk 0.80, 95% confidence interval [CI] 0.77–0.84) and AD (0.86, 95% CI 0.80–0.93) among more active participants (8). These population-level associations support PA promotion for general and brain health, but they do not identify biological AD in an individual.

Reverse causation is a persistent problem. In the 28-year Whitehall II cohort, midlife PA was not associated with subsequent dementia, whereas activity declined during the years preceding diagnosis (9). Low activity in later life can therefore be an exposure, an early consequence of disease, or both. Etiologic, prognostic, and diagnostic questions require different time origins and analyses. The World Health Organization (WHO) target of 150–300 min of moderate-intensity activity, or 75–150 min of vigorous activity, each week is a public-health recommendation, not an AD-specific threshold (10).

### 4.2 Objective activity, gait, and rest-activity measures

Wearable devices can capture total activity, step count, intensity distribution, sedentary fragmentation, day-to-day variability, timing, and regularity. Instrumented gait adds measures of speed, stride-time variability, turning, and dual-task cost. Objective recording reduces reliance on recall, and repeated measurements allow within-person change to be studied, but the apparent precision of a digital trace can be misleading. Device placement and firmware, wear-time algorithms, season, occupation, built environment, pain, cardiopulmonary disease, sleep disorders, and medication all influence the signal.

In 716 older adults without dementia, actigraphy-derived total daily activity was associated with incident AD and cognitive decline (11). In 78,430 UK Biobank participants, accelerometer-derived step counts were nonlinearly associated with incident all-cause dementia over a median follow-up of 6.9 years (12). Neither study used AD-specific biomarker outcomes. Cross-sectionally, gait variability has been associated with amyloid burden in cognitively unimpaired older adults, but the sample was modest, and the measure is vulnerable to musculoskeletal and vascular confounding (13). These observations identify behavioral measures for further investigation but do not validate their use to select individuals for AD-biomarker testing.

### 4.3 Evidence anchored to amyloid, tau, and cognition

Rest-activity rhythm studies show both promise and nonspecificity. In a cross-sectional study of 189 cognitively normal adults, greater intradaily variability—more fragmented activity over 24 h—was associated with positive Pittsburgh compound B imaging and with a higher CSF p-tau/Aβ42 ratio after adjustment for age and sex (14). In the Rotterdam Study, baseline 7-day actigraphy in 319 adults without dementia preceded amyloid PET by a mean of 7.8 years. Higher intradaily variability was associated with greater subsequent amyloid burden (standardized β 0.15, 95% CI 0.04–0.26), particularly among apolipoprotein E (APOE) ε4 carriers; other objective and self-reported sleep measures were not associated with amyloid (15). The prospective interval strengthens temporality, but selection for PET and residual confounding limit causal interpretation.

Pedometer studies in the Harvard Aging Brain Study (HABS) examine whether PA is associated with subsequent disease trajectories in relation to previously measured amyloid burden. Among 182 clinically normal older adults, greater baseline PA attenuated amyloid-related cognitive decline and gray-matter volume loss (16). A later analysis of 296 cognitively unimpaired participants extended follow-up and incorporated longitudinal tau PET in 172. Higher baseline step count was associated with slower amyloid-related inferior temporal tau accumulation and slower cognitive and functional decline, but not with lower baseline or longitudinal amyloid burden (17). Exploratory dose–response analyses plateaued at approximately 5,001–7,500 steps per day among participants with elevated amyloid. This range should not be reinterpreted as a screening cut point or treatment prescription: PA was measured only at baseline, the cohort was selective, the study was observational, and key significance testing was not adjusted for multiple comparisons.

These two analyses (16, 17) draw on the same cohort at different follow-up stages and should not be treated as independent replications. To our knowledge, no independent cohort has yet replicated the step-count–tau association among biomarker-characterized cognitively unimpaired adults. Furthermore, PA was measured only at baseline, the cohort was demographically selective, and no incremental-value analysis relative to simple clinical predictors was reported.

A 2025 Korean multicenter study offers a useful counterweight. Among 1,144 participants spanning cognitively unimpaired, mild cognitive impairment, and dementia, higher self-reported PA was associated with lower plasma p-tau217 and neurofilament light and with better cognition (18). However, within the cognitively unimpaired subgroup, trends across PA quartiles were not significant for Aβ42/40, glial fibrillary acidic protein, neurofilament light, cognition, or p-tau217; only the highest PA quartile had lower p-tau217 than the lowest. The study was cross-sectional, and PA was self-reported. Thus, mixing clinical stages can yield an overall association that does not reproduce in the specific preclinical target population.

### 4.4 Intervention evidence and mechanistic interpretation

PA may influence brain aging through vascular and metabolic health, neurotrophic signaling, sleep and circadian regulation, inflammation, and reserve. A randomized trial in healthy older adults found that 1 year of aerobic exercise increased anterior hippocampal volume by about 2% and improved spatial memory (19). Cross-sectional studies have also reported associations between habitual exercise and CSF or amyloid PET measures (20, 21). These results establish plausibility, not surrogate validity.

The most direct intervention test has been less encouraging for amyloid. In a 52-week trial, 117 cognitively normal older adults with elevated or subthreshold amyloid were randomized to 150 min per week of aerobic exercise to an education control (22). Fitness improved by 11% in the exercise group compared with 1% in controls, but amyloid accumulation, brain volume, and cognitive change did not differ. A true effect may require a larger sample or longer exposure, but the null result does not support the claim that exercise reliably lowers amyloid over 1 year. Benefits of PA may operate through vascular, resilience, tau-related, or non-AD pathways even when amyloid is unchanged (see Table 1).

**Table 1:** Representative human evidence linking physical activity or rest-activity measures to dementia-related or AD-specific outcomes.

Study and design	Population and exposure	Outcome and main finding	Interpretive limit	Incremental predictive comparison beyond clinical predictors
Buchman et al., 2012 (11) Prospective cohort	716 older adults without dementia; actigraphy-derived total daily activity	Lower total activity was associated with incident AD and faster cognitive decline.	No AD-specific biomarker; reverse causation and comorbidity remain plausible.	Not reported
del Pozo Cruz et al., 2022 (12) Prospective cohort	78,430 UK Biobank participants; wrist-accelerometer step metrics	Step count and intensity showed nonlinear associations with incident all-cause dementia over a median of 6.9 years.	Registry-based dementia; no amyloid, tau, or CSF reference outcome.	Not reported
Musiek et al., 2018 (14) Cross-sectional	189 cognitively normal adults; 7–14 days of actigraphy	Rest-activity fragmentation was associated with PiB positivity and the CSF p-tau/Aβ42 ratio.	Cross-sectional and modest sample; fragmentation is not AD-specific.	Not reported
Rabin et al., 2019 (16) Longitudinal cohort	182 clinically normal HABS participants; baseline pedometer steps	Higher PA attenuated amyloid-related cognitive decline and gray-matter volume loss.	Single baseline PA assessment; selected observational cohort.	Not reported
Vidoni et al., 2021 (22) Randomized trial	117 underactive cognitively normal adults with elevated or subthreshold amyloid; 52-week aerobic exercise vs. education	Fitness improved, but changes in amyloid, brain volume, and cognition did not differ between groups.	One year and sample size may be insufficient for slow biomarker change.	Not assessed
Nguyen Ho et al., 2024 (15) Prospective cohort	319 adults without dementia; baseline 7-day actigraphy; mean 7.8-year interval to amyloid PET	Greater intradaily variability was associated with higher later amyloid burden; other sleep measures were null.	PET subset selection and residual confounding; the association in the fully adjusted model did not remain significant after FDR correction (*p* = 0.12).	Not reported
Kim et al., 2025 (18) Cross-sectional	1,144 participants across the cognitive spectrum; self-reported PA	Overall associations with lower p-tau217/NfL and better cognition were not consistently present in the cognitively unimpaired subgroup.	Mixed clinical stages, self-report, and cross-sectional design.	Not reported
Yau et al., 2025 (17) Longitudinal cohort	296 cognitively unimpaired HABS participants; baseline pedometer; longitudinal tau PET in 172	Higher PA was associated with slower amyloid-related tau, cognitive, and functional change, but not lower amyloid burden.	Single PA baseline, selective cohort, observational analyses, and multiple testing.	Not reported

Incremental predictive value refers to a comparison of predictive performance with and without PA or rest-activity features beyond a specified clinical comparator. “Not reported” indicates the absence of such a reported comparison, rather than evidence that adding PA provides no benefit. Covariate-adjusted associations do not by themselves establish improved individual prediction. The intervention trial evaluated exercise effects rather than incremental prediction.

Studies are representative rather than exhaustive. Rabin et al. (16) and Yau et al. (17) report analyses from the same HABS cohort and are not independent replications.

AD, Alzheimer disease; Aβ, amyloid-beta; CSF, cerebrospinal fluid; FDR, false discovery rate; HABS, Harvard Aging Brain Study; NfL, neurofilament light; PA, physical activity; PET, positron emission tomography; PiB, Pittsburgh compound B; p-tau, phosphorylated tau.

Studies were selected to represent the main evidence types (population cohorts, biomarker-anchored cohorts, randomized trials) and are not exhaustive. The scope of study selection is described in Section 2.2. Additional studies discussed in the narrative synthesis are summarized in Supplementary Table S2.

Table 2 summarizes the measurement domains and their proposed roles in this research setting. Gait findings require particular caution because musculoskeletal and neurological disorders can also affect walking performance (23).

**Table 2:** Candidate measures and their roles in biomarker-characterized longitudinal research.

Domain	Examples	Proposed role in this review	Main limitations and interpretation
Wearable PA	Step count and stepping intensity (12, 17).	Behavioral characterization and analysis of associations with subsequent cognitive, functional, and tau changes (16, 17).	Short baseline assessments may not represent activity throughout follow-up. Reverse causation remains possible in observational studies (9, 16, 17).
Gait and performance	Gait speed, variability, turning, and performance during dual-task walking (13, 23).	Characterization of mobility and motor function; investigation of associations with AD biomarkers (13, 23).	Pain, osteoarthritis, and neurological disorders can alter gait (23). Gait abnormalities are therefore not specific to AD (3, 23).
Rest-activity rhythm	Timing, amplitude, interdaily stability, and intradaily variability (14, 15).	Characterization of daily activity organization and investigation of associations with amyloid-related measures (14, 15).	Noncircadian behavior and missing wear data affect interpretation. Rhythm changes also occur with aging independently of amyloid pathology (14).
Clinical context	Age, sex, education, vascular risk, cognition, depressive symptoms, and APOE genotype (15–17).	Clinical characterization, covariate adjustment, and sensitivity analyses of behavioral associations (15–17).	Covariate adjustment does not establish causality. Selective recruitment can limit generalizability (9, 16, 17).
Core biomarkers	Plasma or CSF amyloid/tau measures; amyloid and tau PET (3, 6).	Biological characterization and biomarker outcomes specified by the parent research protocol (3).	Interpretation depends on the assay and clinical setting (3, 6). Disclosure requires consideration of prognostic uncertainty and potential psychological and social consequences (4). Routine testing of cognitively unimpaired individuals outside research is not recommended in the cited guidance (3, 4).

The proposed roles reflect the authors’ synthesis of the cited literature for longitudinal research in biomarker-characterized cohorts. They should not be interpreted as validated clinical indications. Associations involving behavioral measures do not establish causal effects or incremental predictive value. Biomarker acquisition and disclosure remain governed by the approved parent research protocol. AD, Alzheimer disease; APOE, apolipoprotein E; CSF, cerebrospinal fluid; PA, physical activity; PET, positron emission tomography.

## 5 Multimodal analysis: research questions and analytical considerations

### 5.1 Intended use, target population, and outcome

The primary purpose considered here is longitudinal behavioral phenotyping in biomarker-characterized research cohorts. Relevant outcomes include subsequent cognitive and functional change and, when measured under the parent protocol, tau accumulation. The main question is whether PA is associated with these trajectories and whether the associations vary by baseline amyloid burden.

PA may reflect vascular and metabolic health, functional reserve, or circadian organization (14–17, 19). These pathways provide a rationale for studying disease trajectories without implying that PA is specific for AD pathology. They may also overlap with information already captured by clinical variables. Biological plausibility and adjusted associations therefore support investigation but do not establish incremental predictive value.

If individual prediction is examined, a model containing clinical information and relevant baseline biomarkers should be compared with an otherwise comparable model that also includes PA. Both models must predict the same future outcome using information available at the same prediction time and be evaluated on the same independent data. This comparison tests whether PA adds predictive information; it does not assume that it does.

### 5.2 What existing multimodal models establish

Multimodal deep-learning systems have shown that MRI, cognition, demographics, genetics, and clinical information can support AD assessment (24). These studies largely address diagnostic or mixed-stage populations and do not provide evidence that free-living activity improves community enrichment.

A 2025 framework trained on seven cohorts comprising 12,185 participants estimated amyloid and tau PET status from demographics, health history, genetics, neuropsychological tests, physical and neurological examinations, and multisequence MRI (25). Reported AUROCs were 0.79 for amyloid and 0.84 for tau, with evaluation in held-out and external datasets. This is an important proof of concept for multimodal fusion. It did not use free-living wearable PA; however, its cohorts, feature availability, and clinical composition differ from a community sample of cognitively unimpaired adults. The study therefore demonstrates the feasibility of multimodal fusion but does not evaluate free-living PA and provides no evidence for the incremental value of activity data.

Recent studies on wearable sensing show how behavioral signals can be processed for cognitive assessment. Yang et al. (26) combined inertial and plantar pressure signals in a fusion model for cognitive impairment classification. Seo et al. (27) used smartphone and smartwatch gait data collected during daily life to distinguish participants with mild cognitive impairment or mild AD from those with subjective cognitive decline. PreAlzNet explored attention-based sequence modeling of smartwatch data (28). These approaches provide methodological examples, but their relevance to longitudinal research depends on the population studied, the outcome definition, and the timing of assessment.

Rao et al. (29) reviewed gait-based AI methods across neurodegenerative diseases, placing individual modeling approaches within a broader account of sensing technologies and analytical methods. AlzDetect examined heterogeneous clinical and wearable-derived datasets through separate dataset-specific evaluations (30). Such work can inform the choice of measurements and analytical methods for future studies. However, its reported classification performance should not be interpreted as evidence that PA improves prediction in biomarker-characterized, cognitively unimpaired adults. That question requires a direct comparison of models with and without PA, using the same clinical and baseline biomarker information and independent evaluation data.

### 5.3 Architecture, missing modalities, and leakage

For the primary association question, prespecified longitudinal models should account for repeated observations, relevant covariates, and, where justified, interactions among PA, baseline amyloid burden, and time. For an optional prediction task, parsimonious models using interpretable behavioral summaries should serve as benchmarks. More complex AI methods may be evaluated when the sample size and data structure justify their use. No specific architecture is required, and additional complexity should not be treated as evidence of predictive benefit.

Device and sensor variability require explicit statistical handling. Practical strategies include: (i) treating device brand, model, and firmware as stratification or random-effect variables; (ii) harmonization approaches such as ComBat, covariate adjustment, and transfer learning; (iii) overlapping-wear calibration and domain adaptation to reduce device-specific signal; (iv) adjusting for season, wear time, and day-of-week using mixed-effects or temporal-normalization models; and (v) explicit non-wear detection and reporting. During prediction-model development, any harmonization or normalization parameters estimated from the data should be fitted within each training resample and then applied to the corresponding evaluation data.

Missing modalities are part of the use case, not a nuisance to hide. Complete-case analysis may select healthier and wealthier participants, whereas indiscriminate imputation can produce biologically implausible values. Sensible options include modality-specific experts, masked training, multiple imputation for suitable tabular variables, and explicit evaluation of common missingness patterns. A missingness indicator may improve prediction because access to testing is socially patterned; it should not be interpreted as biology without investigation.

For predictive analyses, data-dependent preprocessing, feature selection, imputation, representation learning, and threshold tuning should be fitted within the training resample to prevent leakage into the evaluation data. Participant overlap, related individuals across folds, repeated visits split across sets, or use of post-outcome information can inflate performance. Site, scanner, device brand, firmware, season, and recruitment route may serve as shortcuts. Therefore, temporal and geographic external validation is more informative than another random split of the same cohort.

### 5.4 Evaluation, reporting, and fairness

Association analyses should report effect estimates, confidence intervals, covariate definitions, and sensitivity analyses addressing attrition and reverse causation. For an optional prediction analysis, evaluation must match the outcome. For continuous outcomes, such as future cognitive change, assessment of prediction error and calibration in independent data is required. Models with and without PA should be compared directly, with uncertainty reported for performance differences. AUROC and threshold-based measures apply to separately defined classification tasks. Decision-curve analysis is appropriate only when a specific decision and its consequences have been specified.

For prediction-model studies, TRIPOD+AI provides reporting guidance (31), and PROBAST+AI supports the assessment of model quality, risk of bias, and applicability (32). Performance and calibration warrant examination across age, sex, education, socioeconomic context, rural or urban residence, device access, APOE status, and major vascular or musculoskeletal comorbidity. Evaluation should also assess participation, missingness, and outcome-appropriate performance across relevant population groups.

## 6 A framework for longitudinal research in biomarker-characterized cohorts

### 6.1 Cohort definition and behavioral measurement

Figure 1 starts with cognitively unimpaired participants whose biomarker status has been characterized under an approved research protocol independently of PA-based selection. Biomarker-negative comparison participants may be included when appropriate to the question. Align PA, gait, and rest-activity measurements with clinical and biomarker assessments and interpret them alongside demographics, cognition, vascular risk, sleep, mood, medication, and mobility limitations. Repeated behavioral measurements are desirable where available.

**Figure 1:**
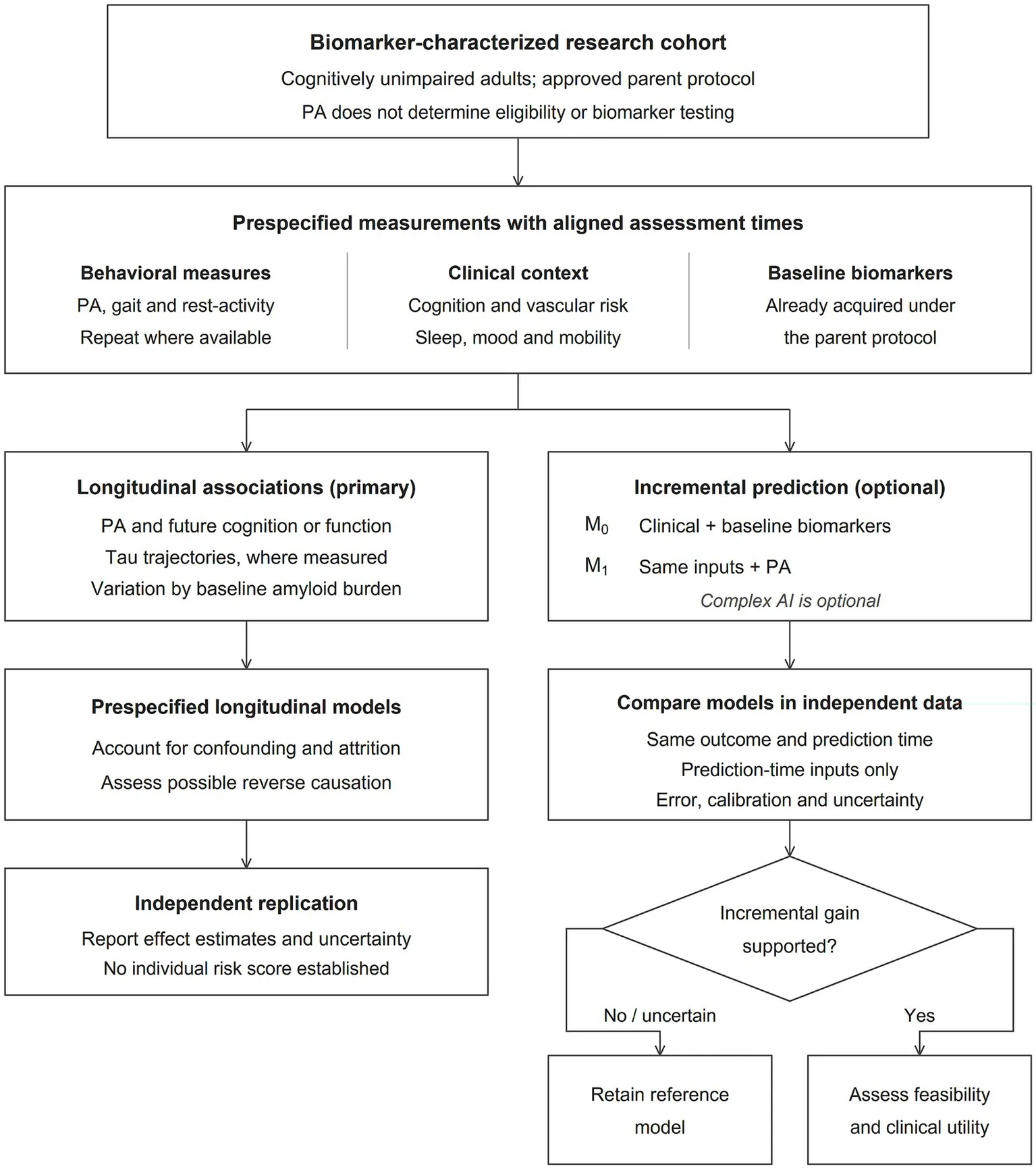
Research framework for PA in biomarker-characterized longitudinal cohorts. Biomarker characterization is determined by an approved parent protocol independently of PA-based selection. The primary analysis examines behavioral associations with subsequent cognitive, functional, and, where available, tau trajectories. A separate optional analysis evaluates whether PA improves prediction beyond clinical and baseline biomarker information. An association does not establish incremental prediction, and neither branch recommends PA-based biomarker referral or automatic recruitment. PA, physical activity; AD, Alzheimer disease; AI, artificial intelligence.

### 6.2 Longitudinal associations and optional prediction analysis

The primary analysis assesses whether behavioral measures are associated with subsequent cognitive, functional, or tau trajectories, and whether these associations vary by baseline amyloid burden. Outputs are population-level estimates and their uncertainty. Confounding, attrition, reverse causation, and independent replication require explicit consideration. These analyses do not establish a causal effect of changing PA.

A separate optional analysis evaluates whether PA improves prediction beyond clinical information and baseline biomarkers, using the comparisons described in Section 5. Negative or inconclusive findings should be reported without converting an association into a predictive claim. Neither branch issues biomarker-testing invitations or constitutes a recruitment service.

### 6.3 Data resources and replication priorities

Existing cohorts offer complementary rather than interchangeable data. HABS provides detailed longitudinal cognitive and PET data, with objective step count in a subset, but the sample is modest and demographically selective (17, 33). The Alzheimer’s Disease Neuroimaging Initiative provides standardized imaging, fluid biomarkers, genetics, and follow-up, whereas continuous free-living PA is not uniformly available across participants and phases (34). UK Biobank offers wrist accelerometry at scale and linked health outcomes, but most dementia outcomes are not anchored to AD-specific PET or CSF (12, 35). The Chinese Alzheimer’s Biomarker and Lifestyle cohort combines lifestyle data with CSF biomarkers; published PA measures have mainly been questionnaire-based and cross-sectional (36, 37).

These datasets cannot be naively concatenated because participants, devices, assessment times, missingness, and outcome definitions vary. Initial work should use suitable existing datasets to assess the reproducibility of PA-related longitudinal associations. Optional prediction analyses require adequate sample size and independent evaluation. Further data collection, including in Chinese cohorts, should address defined gaps in representation, repeated behavioral measurement, or outcome timing. Biomarker acquisition must remain justified by the parent study rather than by an assumed benefit of PA-based screening.

### 6.4 Ethics and implementation

Wearables can reveal routines, sleep patterns, frailty, and location-linked behavior. Data minimization, encryption, retention limits, role-based access, and consent that distinguishes research analysis from clinical interpretation are necessary. Device ownership and digital literacy may exclude rural, older, or socioeconomically disadvantaged people, leading to biased models and unequal access to downstream assessment.

Return-of-results plans need to distinguish a behavioral risk estimate, biomarker positivity, and prognosis. Protocols should specify whether results will be disclosed, who provides counseling, how incidental findings are handled, and what support is available after disclosure. Participants should be able to decline biomarker disclosure or withdraw without penalty. The present framework does not recommend additional PET or CSF testing based on PA; costs and participant burden must be justified by the parent protocol and the specific research question.

## 7 Research priorities and limitations

This narrative review outlines several priorities for longitudinal research among cognitively unimpaired adults with existing biomarker characterization. These priorities reflect limitations of the studies discussed and relevant methodological guidance; they are not minimum standards established by this review.

Repeated PA measurements may help characterize behavioral change and its temporal relationship to cognition, function, and tau progression. Baseline-only activity measurements in key longitudinal studies limit conclusions about changes in PA during follow-up (16, 17). Assessment schedules could be tailored to the research question, expected rate of change, measurement reliability, and participant burden. The evidence reviewed does not establish a universal assessment frequency or interval. Independent replication in more diverse cohorts would also help determine whether reported associations extend beyond the original study populations.

For studies that also examine prediction, a testable question is whether PA adds information beyond clinical variables and available baseline biomarkers. Models with and without PA could be compared using the same prespecified outcome, prediction horizon, and validation data. Relevant evaluation aspects include prediction error, calibration, uncertainty, and performance across participant subgroups where sample size permits. TRIPOD+AI provides guidance for reporting prediction-model studies (31), while PROBAST+AI supports assessment of model quality, risk of bias, and applicability (32). These documents provide methodological guidance but do not establish the predictive value of PA. The need for a complex AI model remains an empirical question.

These priorities are subject to the limitations of this review. The search was targeted and restricted to English-language publications, study selection was not performed independently or in duplicate, and no formal study-level risk-of-bias assessment or certainty grading was undertaken. Relevant studies may therefore have been overlooked, and the synthesis may be affected by selection and interpretive bias. In particular, the lack of demonstrated incremental value in the studies discussed here does not establish that PA has no predictive value. The proposed research pathway remains a hypothesis-generating proposal whose feasibility and utility require empirical evaluation. The database queries required terms from all three concept groups and may have missed relevant PA–AD studies without AI or prediction terminology.

## 8 Conclusion

In this study, PA, gait, and rest-activity measures describe behavior and function but are not interchangeable with AD-specific biomarkers. The HABS findings suggest an association between baseline PA and subsequent amyloid-related tau, cognitive, and functional trajectories; however, they do not establish PA as a means of selecting individuals for biomarker testing.

The framework proposed here therefore focuses on longitudinal behavioral research in biomarker-characterized cohorts. Multimodal analysis may help examine variation in disease trajectories, while the need for AI and the additional predictive contribution of PA require separate empirical evaluation. An association with disease progression alone does not justify an individual risk score, a biomarker referral, or a recruitment decision. These research priorities should be interpreted in light of the targeted search and the absence of formal risk-of-bias assessment and certainty grading.
