# Supplementary material for: Physical activity in preclinical Alzheimer disease: current evidence and implications for multimodal longitudinal research
Source: Front Neurol. 2026 Oct 29;17:1978500. doi: 10.3389/fneur.2026.1978500 (PMC13756898; doi:10.3389/fneur.2026.1978500)
Supplement: Supplementary file 1 [file Table_1.DOCX]

**Supplementary Table S1. Full search strings for PubMed, Scopus, and IEEE Xplore.**

| **Database** | **Search strategy** | **Filters** | **Search period** |
| --- | --- | --- | --- |
| PubMed | ("Alzheimer disease"[Mesh] OR "Alzheimer's disease"[tiab] OR "preclinical Alzheimer"[tiab] OR "cognitively unimpaired"[tiab] OR "cognitively normal"[tiab] OR "mild cognitive impairment"[tiab] OR dementia[tiab]) AND ("physical activity"[Mesh] OR exercise[tiab] OR accelerometry[tiab] OR accelerometer[tiab] OR pedometer[tiab] OR wearable[tiab] OR "wearable sensor"[tiab] OR gait[tiab] OR "rest-activity"[tiab] OR "rest-activity rhythm"[tiab]) AND ("artificial intelligence"[tiab] OR "machine learning"[tiab] OR "deep learning"[tiab] OR multimodal[tiab] OR prediction[tiab] OR validation[tiab] OR "cohort enrichment"[tiab]) | English; Humans | Inception to 25 August 2026 |
| Scopus | TITLE-ABS-KEY("Alzheimer disease" OR "Alzheimer's disease" OR "preclinical Alzheimer" OR "cognitively unimpaired" OR "cognitively normal" OR "mild cognitive impairment" OR dementia) AND TITLE-ABS-KEY("physical activity" OR exercise OR accelerometry OR accelerometer OR pedometer OR wearable OR "wearable sensor" OR gait OR "rest-activity" OR "rest-activity rhythm") AND TITLE-ABS-KEY("artificial intelligence" OR "machine learning" OR "deep learning" OR multimodal OR prediction OR validation OR "cohort enrichment") | English | Inception to 25 August 2026 |
| IEEE Xplore | ("All Metadata":"Alzheimer" OR "All Metadata":"cognitively unimpaired" OR "All Metadata":"mild cognitive impairment") AND ("All Metadata":"physical activity" OR "All Metadata":wearable OR "All Metadata":gait OR "All Metadata":accelerometer OR "All Metadata":"rest-activity") AND ("All Metadata":"machine learning" OR "All Metadata":"artificial intelligence" OR "All Metadata":multimodal OR "All Metadata":prediction OR "All Metadata":validation) | English | Inception to 25 August 2026 |

**Supplementary Table S2. Additional studies discussed in the narrative synthesis.**

| **Study** | **Design** | **Population** | **Exposure** | **Outcome** | **Contextual contribution and limitations** |
| --- | --- | --- | --- | --- | --- |
| Sabia et al., 2017 [9] | Prospective cohort (Whitehall II) | 10,308 civil servants | Self-reported midlife PA | Incident dementia | Long follow-up informs reverse-causation concerns; self-reported exposure and no AD-specific biomarker. |
| Erickson et al., 2011 [19] | Randomized controlled trial | 120 older adults | Aerobic exercise | Hippocampal volume; spatial memory | Intervention evidence for brain structure and memory; no dementia or AD-specific biomarker outcome. |
| Tian et al., 2018 [13] | Cross-sectional | 99 older adults without neurological disease | Gait variability | Amyloid burden | Gait-related amyloid evidence; small sample and cross-sectional design. |
| Liang et al., 2010 [20] | Cross-sectional | 69 cognitively normal adults (PiB-PET: n = 54; CSF: n = 56) | Self-reported exercise | Amyloid PET; CSF Aβ42, total tau, and p-tau181 | Exercise-biomarker associations; cross-sectional design and modest sample size. |
| Head et al., 2012 [21] | Cross-sectional | 201 cognitively normal adults aged 45–88 years (PiB-PET: n = 163; CSF: n = 165) | Self-reported exercise engagement | Amyloid PET; CSF Aβ42 | Exercise, APOE genotype, and amyloid-related measures; no longitudinal outcome. |

These studies provide additional context for the narrative synthesis and are not an exhaustive list. Reference numbers correspond to the main manuscript. PET and CSF sample sizes describe modality-specific subsets and must not be added together.

Abbreviations: AD, Alzheimer disease; Aβ, amyloid-beta; APOE, apolipoprotein E; CSF, cerebrospinal fluid; PA, physical activity; PET, positron emission tomography; PiB, Pittsburgh compound B; p-tau, phosphorylated tau.
